# In-Vitro Catalytic and Antibacterial Potential of Green Synthesized CuO Nanoparticles against Prevalent Multiple Drug Resistant Bovine Mastitogen *Staphylococcus aureus*

**DOI:** 10.3390/ijms23042335

**Published:** 2022-02-20

**Authors:** Anwar Ul-Hamid, Hatim Dafalla, Abbas Saeed Hakeem, Ali Haider, Muhammad Ikram

**Affiliations:** 1 Core Research Facilities, King Fahd University of Petroleum & Minerals, Dhahran 31261, Saudi Arabia; dmhatim@kfupm.edu.sa; 2 Interdisciplinary Research Center for Advanced Materials, King Fahd University of Petroleum & Minerals, Dhahran 31261, Saudi Arabia; 3 Interdisciplinary Research Center for Hydrogen and Energy Storage, King Fahd University of Petroleum & Minerals, Dhahran 31261, Saudi Arabia; ashakeem@kfupm.edu.sa; 4 Faculty of Veterinary and Animal Sciences, Muhammad Nawaz Shareef University of Agriculture (MNSUA), Multan 66000, Pakistan; ali.haider@mnsuam.edu.pk; 5 Solar Cell Applications Research Lab, Department of Physics, Government College University, Lahore 54000, Pakistan; dr.muhammadikram@gcu.edu.pk

**Keywords:** bactericidal potential, CuO, nanoparticles, dye degradation, MDR

## Abstract

Nanoparticles prepared from bio-reduction agents are of keen interest to researchers around the globe due to their ability to mitigate the harmful effects of chemicals. In this regard, the present study aims to synthesize copper oxide nanoparticles (CuO NPs) by utilizing root extracts of ginger and garlic as reducing agents, followed by the characterization and evaluation of their antimicrobial properties against multiple drug resistant (MDR) *S. aureus*. In this study, UV-vis spectroscopy revealed a reduced degree of absorption with an increase in the extract amount present in CuO. The maximum absorbance for doped NPs was recorded around 250 nm accompanying redshift. X-ray diffraction analysis revealed the monoclinic crystal phase of the particles. The fabricated NPs exhibited spherical shapes with dense agglomeration when examined with FE-SEM and TEM. The crystallite size measured by using XRD was found to be within a range of 23.38–46.64 nm for ginger-doped CuO and 26–56 nm for garlic-doped CuO. Green synthesized NPs of ginger demonstrated higher bactericidal tendencies against MDR *S. aureus*. At minimum and maximum concentrations of ginger-doped CuO NPs, substantial inhibition areas for MDR *S. aureus* were (2.05–3.80 mm) and (3.15–5.65 mm), and they were measured as (1.1–3.55 mm) and (1.25–4.45 mm) for garlic-doped NPs. Conventionally available CuO and crude aqueous extract (CAE) of ginger and garlic roots reduced MB in 12, 21, and 38 min, respectively, in comparison with an efficient (100%) reduction of dye in 1 min and 15 s for ginger and garlic doped CuO NPs.

## 1. Introduction

Bovine mastitis is represented by the inflammation of mammary gland parenchyma. Across the global dairy industry, this endemic disease affects lactating animals. It has emerged as a serious malaise that threatens to jeopardize the farm economy [1,2]. Among one hundred various micro-organisms related to bovine mastitis, the most common are *Staphylococci* and Gram-negative bacteria [3,4,5]. Moreover, Gram-positive and negative bacteria represent a big threat to the community’s well-being due to the proliferation of numerous drug resistant bacterial strains [6,7,8]. *Staphylococcus aureus* (*S. aureus*) is a common pathogenic bacterium responsible for up to 40% of mastitis cases in dairy animals [9]. Commonly used treatments for mastitis include β-lactam antibiotics in dairy animals [10]. Irrational use of antibiotics results in the development of antibiotic resistance in bacteria [11]. The major etiology *S. aureus* for chronic, sub-clinical and clinical mastitis is challenging for all types of treatments [12]. Numerous genetic capabilities endorse the pathogenicity of microorganisms, and the methicillin-resistant *S. aureus* (MRSA) isolates boost its pathological processes in mastitis by eluding the host’s immune responses [13,14]. MRSA has been declared a threat to animals and humans [15,16]. The zoonotic potential of MRSA includes direct contact with animals, contaminated environments, and fomites [17] as infectious diseases, which are major threats to the livestock industry around the globe [18,19,20].

Multiple drug resistant (MDR) *S. aureus* infections result in huge economic losses and high morbidity, as this organism is commonly found in bovine raw milk, which is considered a major etiology of bovine mastitis as humans also consume raw milk [21,22]. Raw milk and its products are reported as a major source of *S. aureus* spread [23]. *S. aureus* infections commonly lead to dermatitis, pneumonia, and septicemia [8]. *S. aureus* contains various types of virulence factors which develop silently and generate toxins. Among these are leukotoxins as leukocidin GH, Panton-Valentine leukocidin or leukocidin DE, as well as modulins of type α, γ-hemolysin and δ-toxins [24,25,26,27,28]. After the pathogen enters the skin of the host, an inflammatory response occurs, and host cells enfold the pathogen to prevent the formation of specific structures [29].

The latest advancements in nano-biotechnology have contributed towards the production of new antimicrobial agents. In particular, this includes the synthesis of metal oxide nanomaterials bearing a unique shape and size. The development of advanced nanomaterials is a major contribution of nanotechnology. Nanoparticles (NPs) are usually <100 nm in size, whereas particles of zero dimension are referred to as quantum dots [30]. The excessive use of antibiotics against pathogenic bacteria is the key underlying factor for the development of multiple resistance genes and pathogen resistance in bacteria [31]. It is estimated that 70% of microbe etiologies are resistant to one or more widely used antibiotics [32]. Due to this reason, advances in the synthesis of effective and novel bactericidal agents carry huge significance [33]. Besides various techniques adopted for the development of NPs, synthesis using natural green sources as reducing agents attained special attention from researchers due to the latter’s eco-friendly nature, non-toxicity, ease-of-use, energy efficiency, and cost-effectiveness [34].

The fabrication of nanoparticles from aqueous extracts of plant sources is a facile process that uses plant extract as a reducing agent and a metallic salt solution [35]. Plant mediated nanoparticles depict a higher bactericidal potential to combat infections in humans, which possess bacterial and fungal etiologies [36]. Various plants, such as *Abies spectabilis* (Himalayan Fir) [37], *Abutilon indicum* (India Abutilon) [38], *Azadirachta indica* (neem) [39], *Banana Peel* [40], *Eucalyptus camaldulensis* (river redgum) [41], *Terminalia phanerophlebia* (Lebombo Cluster-leaf) [42], *Pongamia pinnata* (Karum Tree), *Plectranthus amboinicus* (Mexican Mint), *Justicia adhathoda* (Malabar nut), *Cassia auriculata* (Tanner’s Cassia), *Sedum alfredii Hance* (stonecrop), *Trifolium* (clover)*, Limonia acidissima* (Wood Apple), *Bauhinia racemosa* (bidi leaf tree), *Aspidoterys cordata* (Bokadvel), and *Aloe barbadenis* (aloe vera), have been proclaimed as reducing agents in the green synthesis of NPs [43,44,45]. The plant extract concentration is critical in enhancing the size and morphology of the nanoflowers synthesized using *Nyctanthes arbor-tristis* (Coral Jasmine) and *Calliandra haematocephala* (Red Powder Puff) aqueous extracts [46,47]. The spherical morphology stays uniform as the concentration of plant extract increases, but the size changes. The rising concentration of phytochemical compounds causes a change in the size of the NPs. According to A.P. Angeline Mary and coworkers, increasing the sugarcane juice proportion in the production of CuO NPs from 2 to 10 mL results in a change in the size of the biosynthesized NPs from 29.89 to 23.93 nm [48]. Mahboubeh Kargar and colleagues demonstrated that altering the quantity of plant extract used to generate CuO NPs, such as 20, 40, or 80 mL of sour cherry juice, results in an average particle size of 105, 90, or 70 nm [49]. The aforementioned findings demonstrate unequivocally that increasing the amount of plant extract in the reaction mixture results in a reduction in the size of CuO NPs.

Catalysis has been explored and reported in the literature as a crucial and potentially useful topic for the efficient usage of biosynthetic CuO NPs [50]. For instance, Maham et al. effectively manufactured CuO NPs using Euphorbi chamaesyce leaf extract and studied their catalytic reduction of 4-nitrophenol [51]. They discovered that within 180 s, CuO NPs could achieve 100% 4-nitrophenol removal. Additionally, after four consecutive runs, the conversion was nearly undetectable. Additionally, Nasrollahzadeh et al. and Bordbar et al. discovered that the production of CuO NPs utilizing Gundelia tournefortii and Rheum palmatum L. root extracts may significantly decrease 4-nitrophenol [52,53]. On the other hand, significant research has shown that biosynthetic CuO NPs may be used to remediate the environment from various hazardous compounds and contaminants, such as potassium periodate [54], rhodamine B, and methylene blue [52].

The aim of the present study is to evaluate the catalytic potential and antimicrobial activity of green synthesized nanostructures of CuO in regard to MDR *S. aureus* isolates of bovine mastitis.

## 2. Results

**Figure 1:**
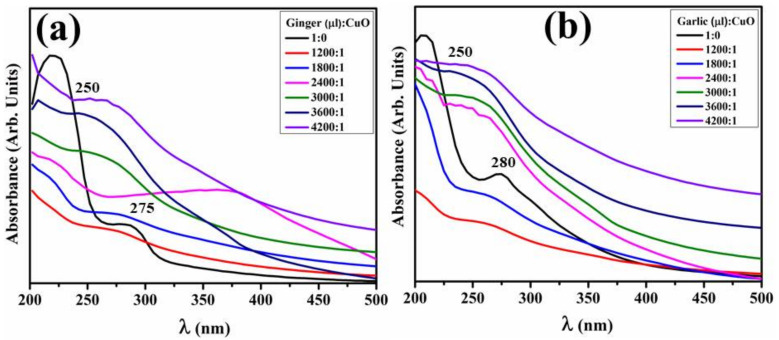
Absorption spectra of (**a**) Gi-doped CuO and (**b**) Ga-doped NPs.

The visual characteristics of green synthesized CuO comprising hydro extracts of Gi and Ga with numerous ratios, i.e., 1200, 1800, 2400, 3000, 3600 and 4200 μL, were measured in the 200–500 nm range, as seen in Figure 1a,b. Absorption peaks of Gi and Ga root extracts presented by 1:0 (extract (μL):CuO) were depicted at 275 and 280 nm, respecitvely. Absorption decreased as the extract amount of CuO increased, whereas the highest absorbance in doped NPs was around 250 nm (3600 μL:1) followed by a redshift in Gi and Ga with the optimized product. This shift is attributed to the surface plasmon absorption of particles [55]. Abrupt color change and absorption at 250 nm indicate particle formation and completion of the reduction reaction. Hence, in Figure 1a,b, results suggest that absorption of synthesized NPs decrease with an increase or decrease in extract volume beyond the optimized ratio (3600 μL:1). 

**Figure 2:**
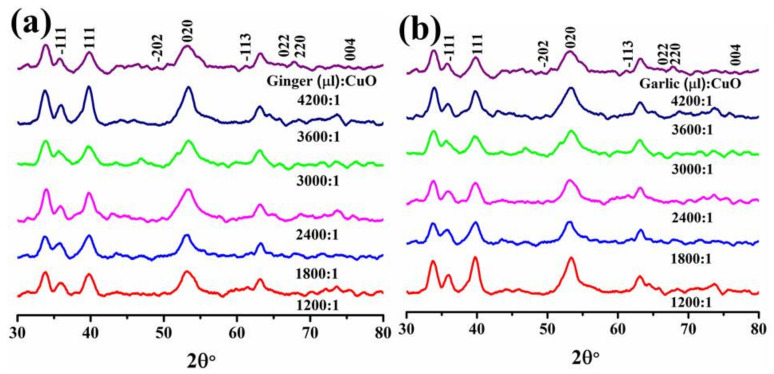
XRD patterns of various concentrations of (**a**) Gi-doped (**b**) and Ga-doped CuO NPs.

As shown in Figure 2a,b, XRD was used to determine crystal size, shape, and crystallinity of doped CuO NPs. XRD data show an increase in crystallinity after doping, with 2θ peak positions at 35.7°, 38.9°, 49.2°, 53.8°, 61.7°, 66.2°, 68.4°, and 75.3° corresponding to crystal planes (−111), (111), (−202), (020), (−113), (022), (220), and (004), respectively. The crystallite size was measured using Scherrer formula D = 0.9λ/βcosθ (where k is shape factor (0.9), θ is peak Bragg’s angle, λ is X-ray radiation wavelength (1.54 A°) and β is the broadening of the diffraction line measured at peak full width of half maximum (FWHM)) and ranged from 23.38 to 46.64 nm for Gi-doped CuO as shown in Figure 2a and 26–56 nm for Ga-doped CuO as shown in Figure 2b [56].

**Figure 3:**
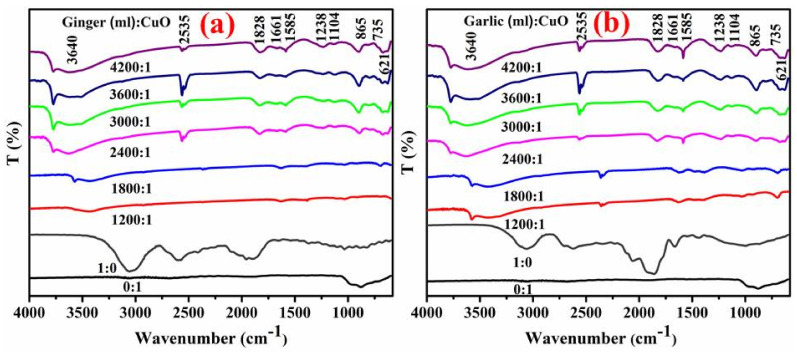
(**a**) FTIR analysis with Gi extract: CuO (**b**) Ga.

FTIR analysis revealed biomolecules as possible functional groups in Gi- and Ga-doped CuO during synthesis, as shown in Figure 3a,b and in Table 1 and Table 2. Observed FTIR bands around 3640, 2535, 1828, 1661, 1585, 1238, 1104 and 865, and 735 and 621 cm^−1^ are associated with O-H, CO_2_ stretching vibrations, C=C stretching of the aromatic ring, the C=O carbonyl group, aromatic ring C-C stretch, aliphatic nitro compounds, and CH functional groups, respectively, at maximum dopant concentrations of root extracts. Similarly, at the lowest concentration of extract dopant, absorption peaks appeared at 701, 1040, 1362, 1633, and 3582 cm^−1^ as depicted in Table 1. These peak shifts with maximum doping of Gi root extracts indicate phytochemicals to be α-Zingeberene, 6-gingerol, and 6-snogal of Gi, which were found to be significant for bio-reduction. In the case of lowest Ga root extract doping, the absorption peaks appeared at 701, 916, 1048, 1362, 1635, 2363, and 3582 cm^−1^ as presented in Table 2. 

**Figure 4:**
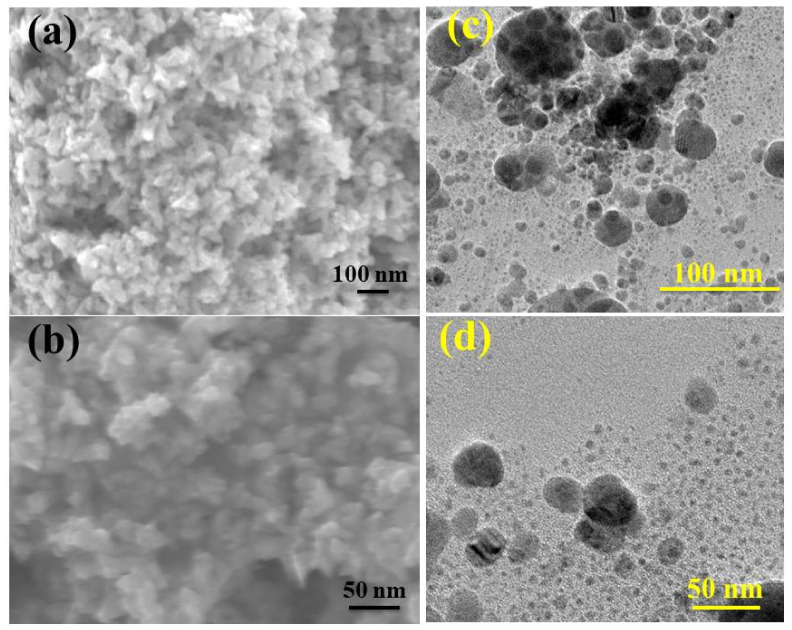
(**a**) SEM depiction of Gi-doped CuO (**b**) Ga-doped CuO (**c**) TEM depiction of Gi-doped CuO (**d**) Ga-doped CuO.

Sample morphology and the size of synthesized NPs were analyzed with the use of scanning and transmission electron (FE-SEM and FE-TEM) microscopes as presented in Figure 4a–d. Green synthesized CuO NPs displayed spherical structures and dense agglomeration, as shown in Figure 4a,b. From FE-TEM images, a high degree of agglomeration of doped NPs was observed. It is noteworthy that the particle size of doped NPs is less than 50 nm, as shown in Figure 4c,d. Dense agglomeration was observed in Ga-doped CuO NPs in comparison with Gi-doped NPs.

**Figure 5:**
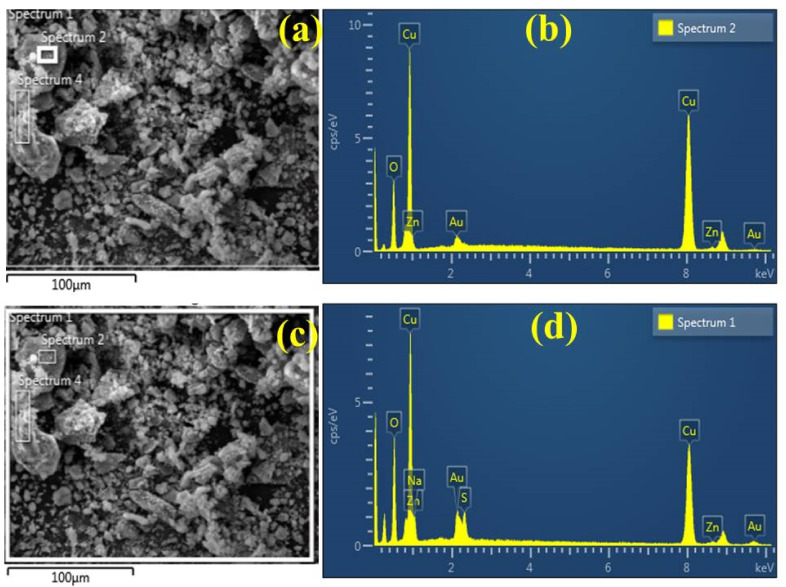
(**a**) SEM image and (**b**) EDS spectrum obtained from fabricated Gi-doped CuO NPs (**c**) SEM image and (**d**) EDS spectrum of Ga-doped CuO NPs.

Energy dispersive X-ray spectroscopy (EDS) combined with SEM was used to investigate the elemental constitution of synthesized NPs. The EDS findingsin Figure 5b confirmed pure Cu phases with distinct peaks within 1 and 10 keV energy range. These peaks specifically indicate highly pure Cu in specimens under study. The atomic weight percentages recorded with the spectra for Cu and O were 82.81% and 15.76%, respectively, for Gi-doped CuO NPs. Figure 5c,d represents the EDS spectrum with an SEM image for Ga-doped NPs with atomic weight percentages of Cu and O as 66.5% and 23.9%, respectively. Additionally, the Au peak seen in Figure 5b is attributed to the Au sputtered coating covering the sample for charge dissipation, and Zn can emanate from the sample holder during SEM examination. In Figure 5d, S corresponds to flavonoids, phenols, and enzymes capping fabricated CuO NPs [57].

**Figure 6:**
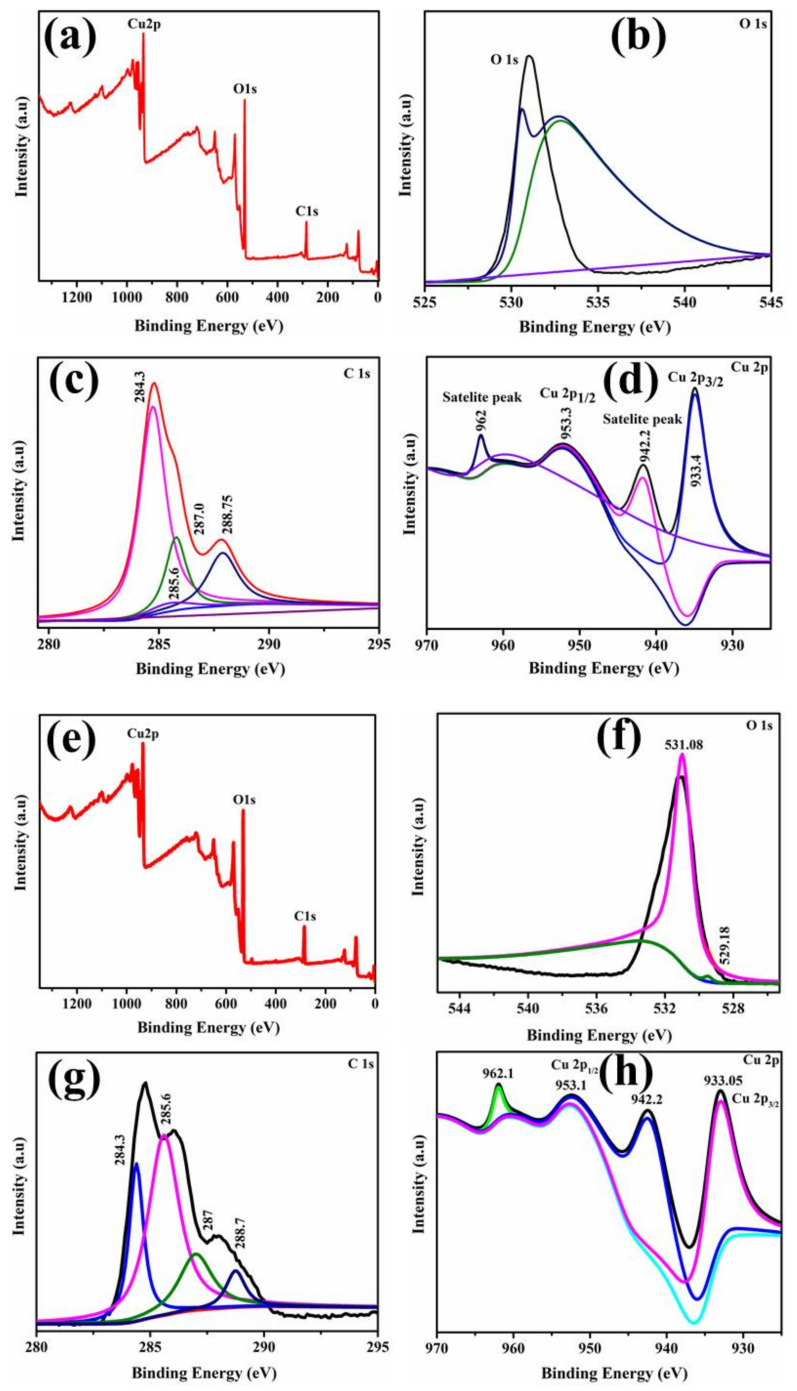
XPS spectra of Gi-doped CuO NPs (**a**) Survey (**b**) O1s orbitals (**c**) C1s spectra of CuO (**d**) Cu 2p (**e**) Survey spectra of Ga-doped CuO NPs (**f**) O1s (**g**) C1s (**h**) Cu 2p.

Surface elemental components as well as surface state and binding energy changes of green synthesized CuO NPs were investigated using XPS. Fabricated samples depicted zero impurities in the XPS survey spectrum, as shown in Figure 6a. The O 1s, C 1s, and Cu 2p high resolution spectrum is presented in Figure 6b–d for Gi and in Figure 6f–h for Ga-doped CuO NPs. The O 1s spectrum depicted binding energies at 529.18 and 531.08 eV for both Gi and Ga doping, as shown in Figure 6b,f, corresponding to CuO lattice oxygen [58,59]. Doped NPs contributed primarily to the C1s band at 284.3 eV through C–C and C(H,C) bonds and C–O (hydroxyl and ether-like) moieties at 285.6 eV as presented in Figure 6c,g. At 287 and 288.7 eV, a significant and stable response of more oxidized species (such as carbonyl and carboxylic) indicates the existence of highly oxidized species, which might be attributable to the presence of C(O, =N), C–O–C, C–OH, and C–N species [60,61,62]. Particularly, Figure 6d shows a Cu 2p pattern of Gi-doped CuO with peak heights at 933.3 and 953.3 eV. The binding energies matched with Cu 2p_3/2_ and Cu 2p_1/2_ spin orbits and indicated the samples’ divalent oxidation state. The other peaks at 942.2 and 962 eV corresponded to satellite heights of Cu 2p_3/2_ and Cu 2p_1/2_, which appeared principally due to the partially filled 3d^9^ orbital in a divalent oxidation state [63]. Similarly, as shown in Figure 6h, for Ga-doped CuO NPs, the binding energies of Cu 2P_3/2_ and Cu 2P_1/2_ were transferred from 933.05 and 953.1 eV to 942.2 and 962.1 eV, respectively. The prominent peak appearing due to fitting for the Cu2P_3/2_ indicates a copper oxidation state [58].

**Figure 7:**
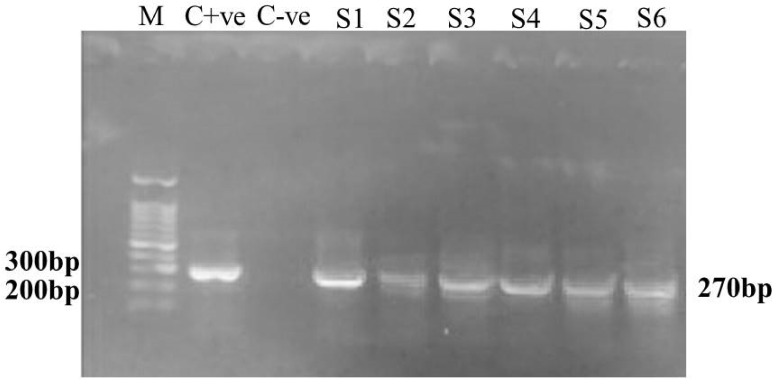
MDR *S. aureus* amplified *nuc*A gene (270 bp) gel picture. Lane M designates 100 bp ladder, C+ve indicates positive control, C—ve shows negative control, S1–S6 indicate MDR *S. aureus* positive isolates from bovine mastitis.

PCR used for the molecular identification of isolated MDR *S. aureus* and *nuc*A gene partial fragments (270 bp) was amplified from 6/10 isolates (Figure 7) originating from bovine mastitis. The PCR result emerged as a single DNA band of about the same size as the 267 bp band from the pBR322 HaeIII DNA digest. This size is comparable to the predicted PCR product value of 279 bp. Repeated testing of many of the strains revealed the same outcome. This observation established the amplified products’ nuc gene identities [64]. 

**Figure 8:**
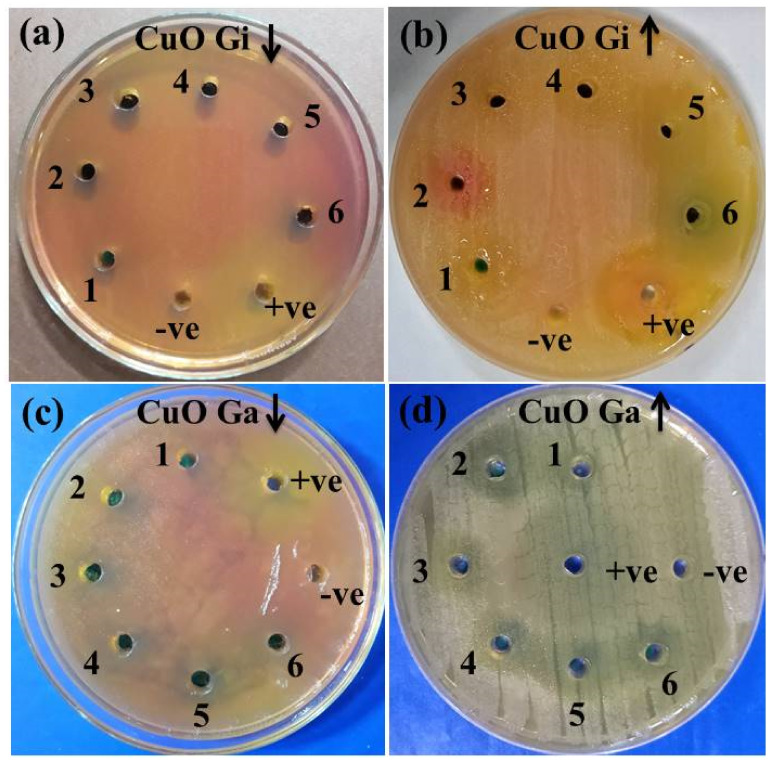
(**a**,**b**) *In vitro* bactericidal potential of CuO NPs doped with Gi extract at minimum (↓) and maximum (↑) doses (**c**,**d**) Ga-doped.

The in vitro antimicrobial efficacy of Gi and Ga root hydro extracts, Copper(II) nitrate, as well as green synthesized CuO NPs, was assessed by resistance areas measurements (mm) using a well diffusion method as demonstrated in Figure 8a–d and Table 3. The findings reveal a clear relationship between the zone of inhibition and concentrations of NPs (*p* < 0.05). Compelling inhibition areas were reported for specimens 1 (1200 μL:1), 2 (1800 μL:1), 3 (2400 μL:1), 4 (3000 μL:1), 5 (3600 μL:1), and 6 (4200 μL:1) against multi-drug resistant (MDR) *S. aureus*ranging (2.05–3.80 mm) and (3.15–5.65 mm) for both minimum and maximum concentrations of Gi*-*doped CuO NPs. These measurements were (1.1–3.55 mm) and (1.25–4.45 mm) for Ga*-*doped NPs. At low concentrations, aqueous extracts demonstrated no efficacy, whereas at maximum concentrations, inhibition areas for Gi and Ga were 1.9 mm and 1.45 mm, respectively. Similarly, Copper(II) nitrate exhibited 1.05 mm and 2.15 mm inhibition areas at minimum and maximum concentrations, respectively, against MDR *S. aureus*. All findings were compared to Ciprofloxacin (7.5 mm) and DW as control positive and negative. In contrast to Ga-doped NPs, green synthesized NPs with Gi dopant showed enhanced bactericidal activity for MDR *S. aureus*.

**Figure 9:**
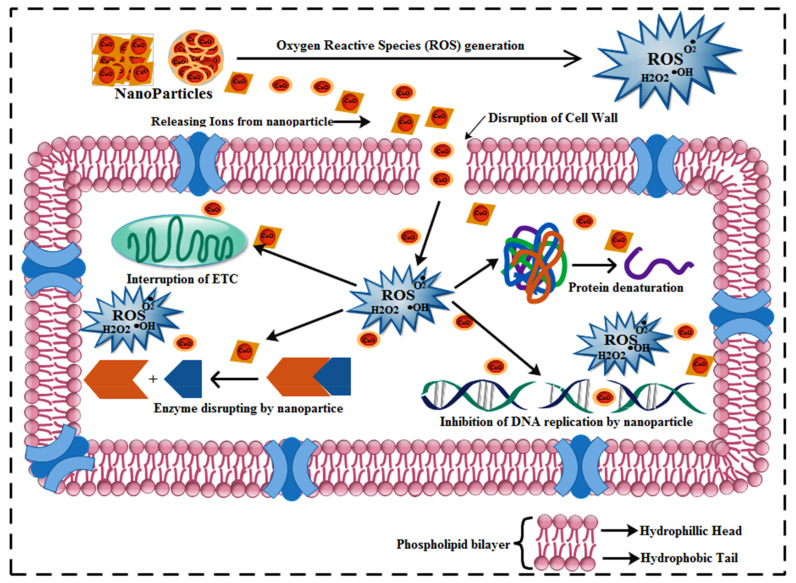
Diagrammatic illustration of the mechanism of antimicrobial activity of garlic and ginger-doped CuO NPs.

The variation in oxidative stress resistance, which is based on several aspects such as shape, composition, and particle size, plays a significant role in a synthesized nanomaterial’s antibacterial potential [65,66]. An electrostatic interface among bacterial strains and nanosized structures involves the production of reactive oxygen species causing cell fatalities [67,68,69,70,71,72]. Two reactions were found to be feasible for a nanomaterial bactericidal mechanism, the first of which is a heavy interaction between cations Cu^2+^ and bacterial cells with negativized sections accompanying collapse, followed by the second, which is a reaction that leads to electronic excitation of CuO valance band surfaces through irradiation. Additional electronic O_2_ reactions produce O^−2^ radicals, leading to the generation of H_2_O_2_. The resulting O^−2^ species have essential roles in the decomposition of the fat or protein molecules in the outer cell surface of the bacteria [73,74], as illustrated in Figure 9.

**Figure 10:**
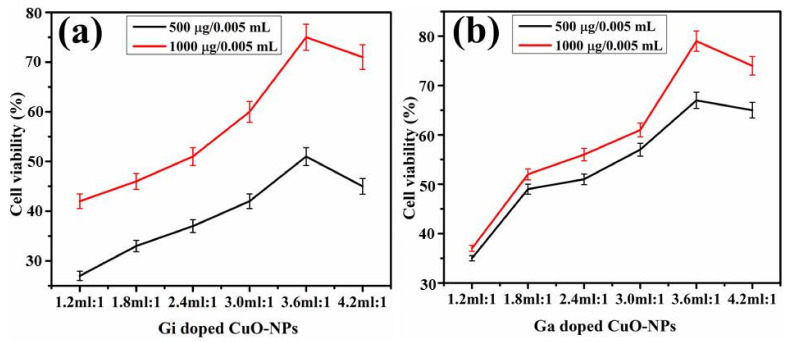
Reduction in percent cell viability of MDR *S. aureus* isolates treated with 500 and 1000 μg/0.05 mL of (**a**) ginger- and (**b**) garlic-doped CuO NPs.

**Figure 11:**
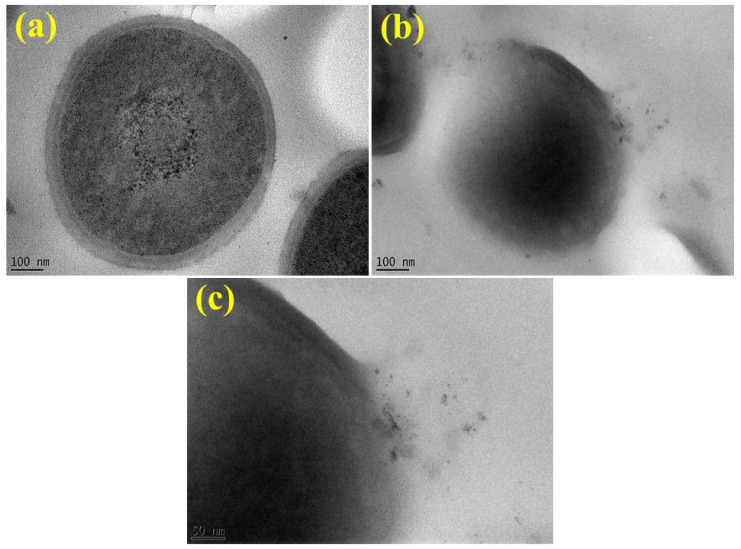
TEM images of (**a**) control MDR *S. aureus* (**b**,**c**) interaction of doped CuO NPs with bacterial cell.

Following 10 h of incubation with doped NPs (500–1000 µg / 0.05 mL), a dosage and time dependent decrease in the proliferative yield (OD620 nm) of MDR *S. aureus* was observed. OD_620_ nm values ranged from 0.47 to 0.19, which are relatively smaller than the OD_620_ nm value of 0.53 for the control samples. Additionally, the bactericidal efficacy of doped CuO NP concentrations (500–1000 µg/0.05 mL) was determined after 24 h of exposure. The percent survival of MDR *S. aureus* was reported to be 49% and 25% for Gi-doped CuO NPs, whereas Ga-doped CuO demonstrated 53% and 41% survival at the minimum and highest concentrations, respectively. At both concentrations, optimized Gi-doped CuO enhanced cell viability by 52% and 76%, which, compared to Ga-doped NPs, enhanced cell viability by 48% and 60%, respectively, as presented in Figure 10a,b. The growth inhibitory findings corroborate the exact patterns of toxicity shown in well diffusion data against MDR *S. aureus*. Similarly, MDR *S. aureus* cells treated with doped CuO NPs produced holes and cavities. The initial spherical form was also destroyed during the contact of NPs with the bacterial cells, as shown in Figure 11b,c.

The TEM study of MDR *S. aureus* treated with doped CuO NPs clearly suggests that NPs are incorporated in substantial amounts, as presented in Figure 11a–c. However, it is not clear if bacterial cells employ an endocytosis mechanism for external entity acquisition. As a result, it is possible that doped CuO NPs infiltrate cells via disruption to the intact cell membrane, resulting in cytoplasmic permeability [75].

CuO NPs were subjected to a molecular docking investigation in order to further understand their mechanistic linkages with the target enzymes. Enzymes involved in the production of peptidoglycans and folic acid are well-characterized, interesting, and plausible targets for antibiotic development. The D alanine-D-alanine ligase (ddlB) is a component of the molecular machinery involved in peptidoglycan manufacturing, and its suppression results in outer membrane rupturing and eventual death. 

**Figure 12:**
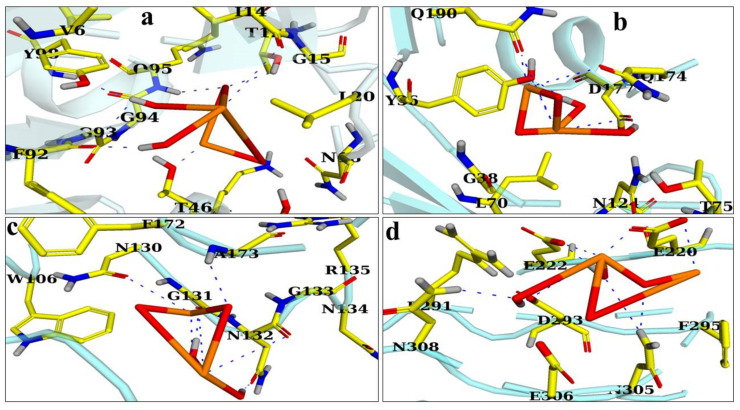
Binding interaction pattern of CuO NPs with active site residues of (**a**) Dihydrofolate reductase, (**b**) Tyrosyl-tRNA synthetase, (**c**) Dihydropteroate synthase, and (**d**) D-alanine-D-alanine ligase from *S. aureus*.

The binding energy of CuO NPs in the active pocket of D-alanine-D-alanine ligase (ddlB) was measured as 6.08 eV. CuO formed H-bonds with Asn308, Asp293, Glu222, Glu220, and Asn305, as illustrated in Figure 12d. Similarly, in the case of tyrosyl-tRNA synthetase, the highest binding score of 6.73 was ascribed to the H-bonding link between Gln190, Tyr36, Asp177, and Gln174, as seen in Figure 12b.

CuO NPs bound to Ile14, Thr12, Gln95, Phe92, Tyr98, Thr46, and Thr121 with a binding index of 5.18, as shown in Figure 12a. Similarly, the best docking score for DHPS from *S. aureus* was 4.08, indicating an H-bonding interaction with Ala173, Gly131, Asn130, and Asn132 (Figure 12c). Table 4 lists the docking scores and critical residues implicated in H-bonding for each protein. The significant binding score and interaction of the CuO NPs suggested that they may be a potential inhibitor of dihydrofolate reductase, D-alanine-D-alanine ligase B (ddlB), tyrosyl-tRNA synthetase, and dihydropteroate synthetase. These NPs can be further investigated for their ability to inhibit enzymes.

### Catalytic Activity

**Figure 13:**
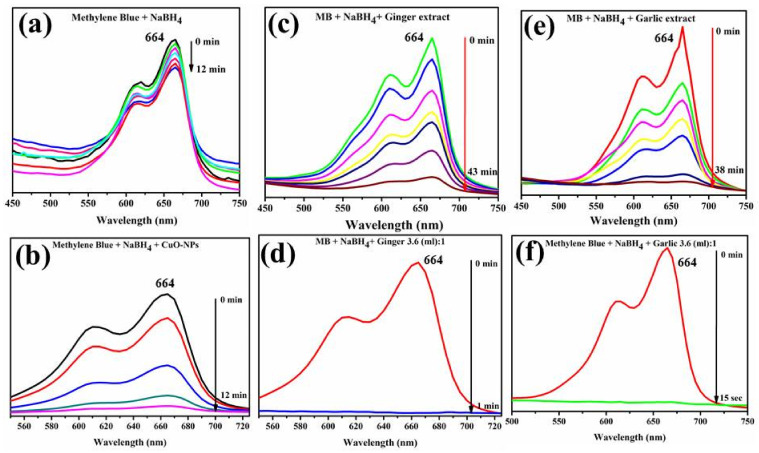
(**a**–**f**) Catalytic response of (**a**) NaBH_4_ (**b**) CuO (**c**) Gi CAE (**d**) Gi-doped CuO (**e**) Ga CAE and (**f**) Ga-doped NPs.

Figure 13a–f shows the dramatic drop in catalytic MB with roots CAE and extract-doped CuO NPs at an ambient temperature. The catalytic action of NaBH_4_ and conventional CuO NPs procured from Sigma-Aldrich is demonstrated in Figure 13a,b, and similarly, the catalytic behavior of CAE of Gi and Gi-doped CuO are shown in Figure 13c,d. The catalytic potential of Ga CAE and Ga-doped CuO NPs is illustrated in Figure 13e,f. A small decrease in absorption peaks was a result of the catalytic action of NaBH_4_ in 12 min, as shown in Figure 13a. Conventionally available CuO and CAE of Gi and Ga roots reduced MB in 12, 43, and 38 min (Figure 13b,c,e, respectively), which lies in comparison with the efficient (100%) reduction of dye in 1 min and 15 s for Gi and Ga extracts doped CuO NPs, as shown in Figure 13d,f. 

## 3. Discussion

In the UV-vis spectrum, a peak positioned at 220 nm indicates the occurrence of polyphenol or aromatic molecular structures [76], and bands at 270 nm arise due to π → π* transitions. These absorption bands depict the existence of phenolic compounds extract [77]. The band at 398 nm could be attributed to smaller sized particles in the CuO nanostructure [78]. The absorption peak shift towards the higher wavelength (redshift) upon doping indicates an increase in particle size and vice versa. The observed peaks in XRD verified the presence of CuO with a monoclinic crystal structure that was well synchronized with JCPDS card number 001-1117 and was free of impurities [79]. 

FTIR bands around 3640, 2535, 1828, 1661, 1585, 1238, 1104, 865, 735 and 621 cm^− 1^ are associated with O-H, CO_2_ stretching vibrations, C=C stretching of aromatic ring, C=O carbonyl group, aromatic ring C-C stretch, aliphatic nitro compound, C–OH, CH functional groups, C–H bends in alkynes and metallic oxygen bonds, respectively, at maximum doping of root extracts [55,71,72,79,80,81]. Similarly, at the lowest concentration of extract doping, absorption peaks appeared at 701, 1040, 1362, and 1633 and 3582 cm^−1^ corresponding to S-O stretching bands, C-N stretch of aliphatic amines, aromatic amines, and C=O and hydroxyl bonds, respectively, as depicted in Table 1. These peak shifts with a maximum doping of Gi root extracts indicate phytochemicals as α-Zingeberene, 6-gingerol, and 6-snogal of Gi, which were found to be significant for bio-reduction. In the case of Ga root extract with the lowest doping, the absorption peaks appeared at 701, 916, 1048, 1362, 1635, 2363 and 3582 cm^−1^, which is equivalent to S-O stretching bands, primary/secondary amines, O-C=O, aromatic amines, moisture content, –C=NH^+^ in charged amines, and hydroxyl-moiety, respectively, as presented in Table 2. Peaks present at low wavelengths between 400–700 cm^−1^ suggest successful synthesis of CuO [82,83]. The Cu and O bond appeared in the form of a sharp and strong band at 584 cm^−1^, indicating the formation of a high purity phase of CuO NPs. The bands at <1000 cm^−1^ are mainly attributed to metal-oxygen bonds [84]. The peak changes in CuO presented by 0:1 (extract (μL):CuO) corresponding to higher amounts of Ga root extracts indicate the presence of allicin (diallylthiosulphinate) and S-allyl cysteine phytoreducers, terpenoids, polyols, flavonoids, and proteins containing ketones and amines accountable for chelation as well as reduction [85,86]. The CuO NPs display stability in water and air and did not transform into other compounds. However, CuO aggregation resulted in a narrow space between NPs during fabrication due to their large surface energy in an aqueous phase [87,88].

The agglomeration of nanostructures occurs as a consequence of the high surface energy of the aqueous medium, which results in a small space between the NPs [87]. Cationic and oligocationic species have an effect on the aggregation of nanoparticles (NPs) in solutions [89]. Aggregation can be prevented by first mixing NPs into foetal bovine serum (FBS) and then into a buffer [90]. NPs may also be prevented from aggregating by covering them with other biomolecules, such as lipids. To enhance NPs’ dispersibility, the first technique is direct physical mixing, in which as-synthesized solid NPs are integrated into the polymer host using a high energy input, such as heat or ultrasound. The enormous energy input disintegrates the bulk material into minute particles [91,92,93].

In the current investigation, we created an oligonucleotide primer set that identified sequences from the *S. aureus* nuc gene, which encodes the bacteria’s TNase. This technique was selected because previous findings obtained by employing polyclonal [94,95] or monoclonal [96] antibodies to identify the *S. aureus* TNase suggested that this protein contains species-specific sequences and that DNA hybridization-based methods confirm this premise [97]. The primer set generated a PCR product with an estimated size of 270 bp, which is similar to the predicted size of 279 bp for the gene fragment. This confirmed that the product is similar to the nuc gene sequence from *S. aureus* [98].

Tyrosyl-tRNA synthetase is an intriguing target enzyme for the discovery of novel antibacterial medicines [99]. It is a member of the family of aminoacyl-tRNA synthetases (aaRSs) and catalyzes the covalent binding of amino acids to their corresponding tRNA to generate charged tRNA. As a result of its critical involvement in protein production, inhibiting aaRSs has a considerable effect on cell proliferation. Folate biosynthesis results in the formation of tetrahydrofolate, which is required for the production of a variety of bioactive compounds, including thymidylate enzyme, pantothenic acid, and nitrogenous bases such as purine, ribonucleic acid, and amino acids. Enzymes involved in this process, such as dihydrofolate reductase and dihydropteroate synthase, have been identified as potential antibiotic targets. Trimethoprim is a well-known antibiotic that inhibits DHFR, whereas the sulfonamide family of medicines inhibits DHPS [100,101].

During catalysis by moving electrons donor (BH_4_) to an acceptor (MB) and decreasing activation energy, the extracts doped NPs cause substantial catalytic color deterioration [102]. MB has the highest absorption peak at 664 nm in an aqueous solution, which is connected to the π→π* and n→π* transitions [103,104]. The data suggested extracts doped NPs as effective catalysts compared with conventional NPs and single CAE.

## 4. Materials and Methods

### 4.1. Materials

Fresh roots of ginger (Gi) and garlic (Ga) were purchased from a local market and shade dried to acquire a consistent weight. Copper(II) nitrate [Cu(NO_3_)_2_], sodium hydroxide (NaOH) and other chemicals of empirical grade were procured from Sigma-Aldrich United States. Media utilized to culture bacteria were of empirical grade.

### 4.2. Aqueous Extracts Preparation

Garlic and ginger powder was produced with an electric grinder, and the resulting fine dust was stored in Ziplock bags. Pulverized root dust was mixed with deionized water and aggressively stirred at 70 °C for half an hour. Crude aqueous extract (CAE) was stored after filtration at 4 °C until further usage [87].

### 4.3. Green Fabrication of CuO NPs

**Figure 14:**
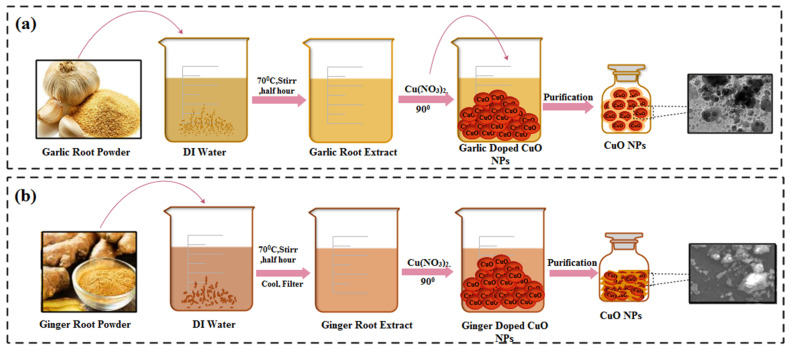
(**a**) Schematic illustration of the procedure followed in the green synthesis of CuO NPs from aqueous extracts of Garlic roots. (**b**) Steps involved in preparation of Ginger-doped CuO NPs.

Numerous ratios of Gi and Ga crude extract (1200, 1800, 2400, 3000, 3600, and 4200 μL) were stirred into 0.1 M (50 mL) copper(II) nitrate at a 90 °C reaction temperature by maintaining the pH at 12 using (2M) NaOH for two hours. The resulting precipitates were washed several times with deionized water (DIW) by undertaking centrifugation for 10 min at 10,000 rpm to remove impurities (nitrates). Obtained pellets were dried in a hot air oven at a constant temperature of 90 °C for 12 h, as shown in Figure 14a,b [46]. Compounds such as phenolic, tannin, saponins, proteins, flavonoids, glycosides and polyphenols are present in the plant material. Additionally, the aqueous extract of these plant materials also contains the chemicals mentioned above, which function as green reductants, reducing Cu^2+^ to Cu^0^ [105,106]. Copper salt forms a polyphenolic compound with Cu^2+^ ions when it is combined with the plant extract. Further reduction results in the conversion of Cu^2+^ to Cu^0^ NPs [50].

#### Characterization of Synthesized CuO NPs

The synthesized NPs were screened with a wavelength of 250–800 nm using a (Genesys 10 S) UV-Visible spectrophotometer to maximize absorption (ƛ_max_). The composition and strucuture of the doped NPs were studied with a BRUKER D2 Phaser (XRD) comprising 2θ range (10–80°) fitted with Cu Kα_1_ radiations of λ = 1.540 Å. The resulting product functionality was checked with Fourier-transform infrared spectroscopy (ATR-FTIR). The elemental constitution of the synthesized metal oxide nanostructures was evaluated through energy-dispersive X-ray spectroscopy. The size and topography of the fabricated nanostructures was confirmed with JEOL FE-SEM and JEM 2100F TEM. The specimen framework with an associated band gap analysis was conducted using X-ray photoelectron spectroscopy (XPS).

### 4.4. Separation and Identification of MDR S. aureus

#### 4.4.1. Collection of Samples

From three private dairy farms located in the districts of Lahore, Kasur, and Sahiwal (Punjab, Pakistan), bovine mastitic fluid specimens were collected as shown in Figure S1.

#### 4.4.2. Isolation of MDR *S. aureus*

The initial culture of the collected samples was carried out on ovine blood agar 5 percent via incubation at body temperature for 48 h. The typical acquired colonies were streaked on mannitol salt agar thrice for the purification of *S. aureus*. The sensitivity of colonies for selected antibiotics was assessed by using a disk diffusion test, as published by the National Committee for Clinical Laboratory Standards (NCCLS) for the degradation of MDR *S. aureus*. Sterile antibacterial disks were used on Mueller–Hinton agar (MHA) containing 1 × 10^8^ CFU/mL growth. The bacteria that were found to be immune to at least three antibacterials were confirmed as MDR after overnight growth at 37 °C [107]. Bacterial patterns were verified using Burgey’s Manual of Determinative Bacteriology, which includes depictions of morphological features and biochemical procedures such as catalase and coagulase tests. Molecular confirmation of MDR *S. aureus nuc*Agene responsible for resistance development was undertaken, and bacterial colonies were processed for DNA extraction using an extraction kit (WizPrepTM gDNA cell/tissue kit, Korea).

#### 4.4.3. Quantification of DNA with a NanoDrop Spectrophotometer

Extracted DNA was quantified with a NanoDrop (Thermoscientific^TM^- NanoDrop2000 Fitchburg, WI, USA). DNA concentration was measured at a 260/280 nm ratio of optical density in ng/μL.

#### 4.4.4. Primers Used and Polymerase Chain Reaction Amplification

A polymerase chain reaction (PCR) test was undertaken for all DNA samples extracted for the *nuc* A gene of *S. aureus* utilizing primers F 5′ -GCGATTGATGGTGATACGGTT-3′ and R 5′ –AGCCAAGCCTTGACGAACTAAAGC-3′ [64] with an amplicon at 267 bp. A total of 3 μL DNA was mixed for PCR reaction mixture preparation. The reaction proceeded at 35 cycles with an initial denaturation at 95 °C for 5 min, annealing at 58 °C with extension at 72 °C. Each step proceeded for half a minute, and the final extension was carried out for 10 min at 72 °C. Obtained PCR products were observed as positive bands (267 bp) by running a 1.5% agarose gel under a UV illuminator using a 100 bp ladder.

### 4.5. Antimicrobial Activity

The assessment of in vitro microbicidal efficacy was carried out with well diffusion tests for ten indicative MDR *S. aureus* isolates. Petri plates were swabbed with 0.5 Mc-Farland of MDR *S. aureus* growth on mannitol salt agar (MSA). Bores having 6 mm width were shaped through clean borer. Different doses of Giand Ga CAE and doped CuO NPs were added. Concentrations of (10,000 and 50,000 µg/100 µL) and (500, 1000 µg/50 µL) were applied for CAE and biosynthesized CuO NPs as minimum and maximum doses, respectively. For comparison, DIW and ciprofloxacin were applied as negative and positive controls with (50 µL) and (5 µg/50 µL) concentrations, respectively. Microbicidal evaluation was undertaken after overnight incubation at 37 °C by inhibition area (mm) measurements through a Vernier caliper.

### 4.6. Effect of Doped CuO NPs on Bacterial Growth and Viability

To accomplish a viability assay, 20 μL of overnight matured MDR *S. aureus* colonies were inoculated onto 96-well microplates. To each well, Luria–Bertani (LB) medium containing a constant amount (300 μL) of Gi- and Ga-doped CuO NPs at 500 and 1000 μg/0.05 mL were loaded. Throughout all the trials, untreated bacteria were incorporated as a control. At 2 h intervals, variations in the optical density at 620 nm (OD_620 nm_) were measured using a microplate reader (Thermo Scientific, Shanghai, China). After 24 h, the percent viability was assessed by comparing the OD_620 nm_ of doped NP-treated bacterial cultures with that of untreated control samples [108].

#### 4.6.1. Pathogen and NPs Interaction with TEM Imaging

Bacterial samples were diluted in PBS and placed on TEM copper grids using a holey carbon sheet in a 10 L specimen drop. After that, the grid was subjected to 70% glutaraldehyde volatiles for 3 h to rectify the samples. To determine the distribution and position of CuO NPs as well as the shape of bacteria treated with doped CuO NPs, strains were studied in a JEOL 2100F TEM in STEM mode using a 200 KV accelerating voltage.

#### 4.6.2. Statistical Analysis

Evaluation of the inhibition areas was carried out using a one-way variance analysis with a 5% significance level through SPSS 20.0.

### 4.7. Molecular Docking Analysis

**Figure 15:**
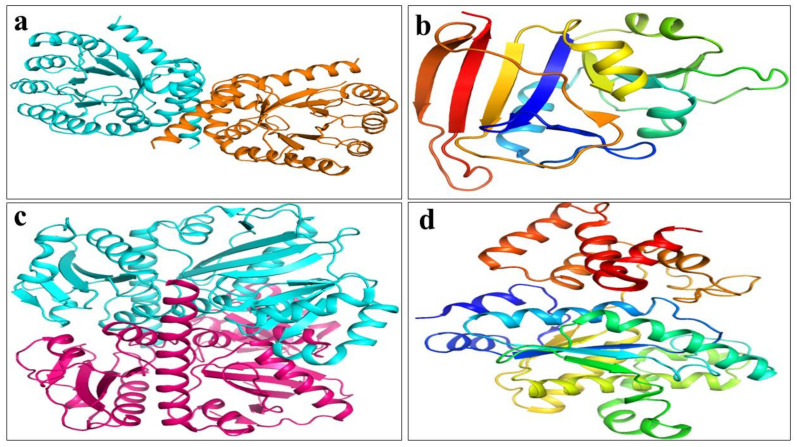
Three-dimensional structures of target proteins of *S. aureus*: (**a**) dihydropteroate synthase (PDB: 4HB7), (**b**) dihydrofolate reductase (PDB: 3FY8), (**c**) D-alanine-D-alanine ligase (PDB: 2I80), (**d**) Tyrosyl-tRNA synthetase (PDB: 1JIJ).

**Figure 16:**
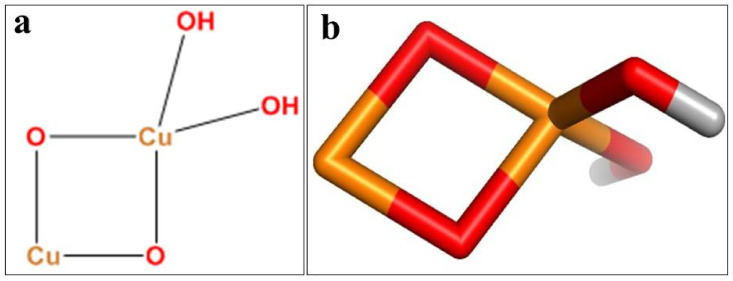
Structure of CuO NPs in (**a**) two-dimensional and (**b**) three-dimensional view.

A molecular docking assessment of fabricated CuO NPs was undertaken to determine their binding affinity with potential targets involved in microbial cell formation. A docking study was performed on a number of protein targets, including dihydrofolate reductase, dihydropteroate synthase, D-alanine-D-alanine ligase B (ddlB), and tyrosyl-tRNA synthetase, as shown in Figure 15a–d. Dihydropteroate synthase and dihydrofolate reductase are essential enzymes in the biogenesis of folic acid and are considered essential for bacteria survival [100]. Equally, the enzymes ddl8 and Tyrosyl-tRNA synthetase act as a catalyst in the biosynthesis of bacteria fatty acids and proteins [109]. The 3D structure of dihydropteroate synthase (PDB ID: 4HB7) with resolution:2.20 Å [110] shown in Figure 15a; relative to dihydrofolate reductase (PDB ID: 3FY8) resolution:1.95 Å [103] is shown in Figure 15b. Similarly, D-alanine-D-alanine ligase B (PDB ID: 2I80) resolution: 2.2 Å [111] is shown in Figure 15c; and that of Tyrosyl-tRNA synthetase (PDB ID: 1JIJ), resolution: 3.2 Å repossessed from a protein data bank as shown in Figure 15d. The molecular docking analysis was conducted with a SYBYL-X 2.0 program [112]. A Sybyl-X2.0/SKETCH module [113] was used to generate 3D structures of the selected compounds followed by energy reduction as per Tripos force fields with atomic loads of Gasteiger–Hückel [114]. To study nanoparticle binding interactions with active site residues of the selected proteins, the Surflex-Dock module for molecular modeling software package SYBYL-X 2.0 was used in versatile molecular docking computations [112]. Missing hydrogen was incorporated. Atomic charges were allocated and applied in accordance with the force field of AMBER 7 FF99 as shown in Figure 16a,b. Finally, energy was reduced to a minimum to avoid steric conflicts by utilizing the Powell method with a 0.5 kcal (mol to A) converging rate for 1000 cycles. At least 20 of the finest docking postures were consistently preserved for each ligand-receptor complex system. The best postures of ligands were measured by adopting the Hammerhead score. By using the Hammerhead scoring function, [115] the best putative postures were measured by ligands [116]. The Surflex-Dock module generates and ranks putative styles of ligand fragments using an empirically derived consensus scoring (cScore) function that integrates Hammerhead’s empirical scoring functions, namely D-score (dock score), G-score (gold score), Chem-Score, potential mean force (PMF) score, and total score, with a molecular similarity method.

### 4.8. Catalytic Activity

**Figure 17:**
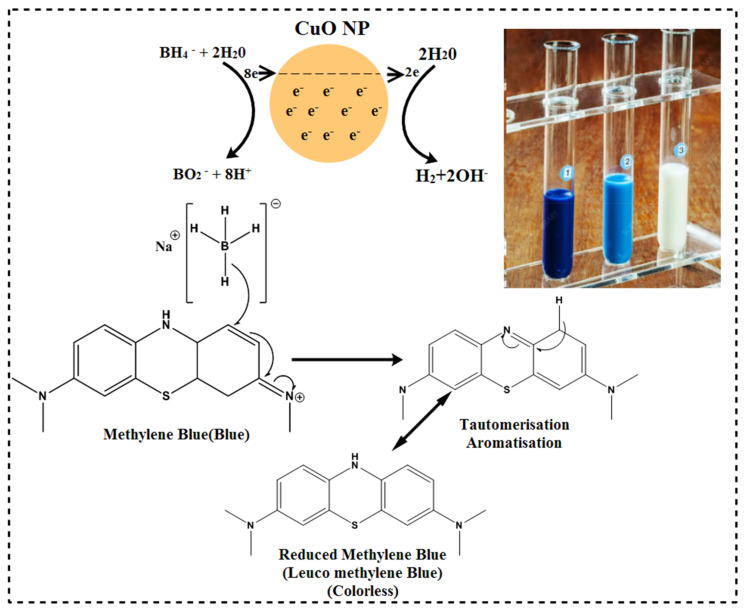
Possible reduction mechanism of methylene blue into leuco methylene blue in the presence of CuO NPs.

For the catalytic assessment of fabricated CuO nanostructures, 3 mL of methylene blue (0.03 × 10^−^^3^ M) was combined with freshly prepared 300 µL sodium borohydride aqueous solution. The optimal value (3600 μL:1) of specimen 6.0 mg/300 µL was consequently applied to the solutions. As a result, the methylene blue (MB) dye color vanished, depicting the deterioration of dye towards leuco methylene blue, as illustrated in Figure 17. A UV-Vis spectrophotometer was used to detect absorption within a 200–800 nm range.

## 5. Conclusions

The current study provides the first report to measure the antibacterial response of doped metal oxide nanostructures against MDR *S. aureus* originating from bovine mastitis. The inclusion of ginger and garlic root extracts in various ratios played a significant role in the fabrication and optimization of metal oxide nanostructures. X-ray diffraction analysis revealed the monoclinic crystal phase of the particles. The calculated crystallite size analyzed with XRD and UV was in the range 23.38–46.64 nm for ginger- and 26–56 nm for garlic-doped CuO. FE-SEM and FE-TEM analysis revealed that the NPs were spherically shaped, exhibiting dense agglomeration. UV-vis spectroscopy revealed a decrease in absorption with an increasing amount of extract in CuO, accompanied with a redshift. The experimental results show that extract-doped NPs were more effective catalysts compared to traditional NPs and single crude aqueous extract (CAE). In comparison to garlic-doped NPs, green synthesized NPs of ginger exhibited enhanced bactericidal potency against MDR *S. aureus*. This study concludes that antibiotic resistance development could be addressed significantly by adopting green synthesized metal oxide nanostructures as antibiotic placebos.

## Figures and Tables

**Table 1:** FTIR spectra of Gi doped CuO NPs.

Sr. No	Absorption Peaks (cm^−1^)	^1^ Functional Moieties/Bond	Absorption Peaks (cm^−1^)	^2^ Functional Moieties
1	701	S-O stretching band	584	Cu and O bond
2	1040	C-N stretch of aliphatic amines	621	C–H bend in alkynes
3	1362	aromatic amines	735	metal-oxygen bonds
4	1633	C=O	865	CH functional groups
5	3582	hydroxyl bond	1104	C–OH
			1238	aliphatic nitro compound
			1585	aromatic ring C-C stretch
			1661	C=O carbonyl group
			1828	C=C stretching of aromatic ring
			2535	CO_2_ stretching vibrations
			3640	O-H

^1^ Functional moieties of Gi-doped CuO NPs at minimum plant concentration. ^2^ Functional groups of Gi-doped CuO NPs at maximum plant concentration.

**Table 2:** FTIR spectra of Ga-doped CuO NPs.

Sr. No	Absorption Peaks	^1^ Functional Bond	Absorption Peaks	^2^ Functional Bond
1	701	S-O stretching band	584	Cu and O bond
2	916	primary/secondary amines	621	C–H bend in alkynes
3	1048	O-C=O	735	metal-oxygen bonds
4	1362	aromatic amines	865	CH functional groups
5	1635	moisture content	1104	C–OH
6	2363	–C=NH^+^ in charged amines	1238	aliphatic nitro compound
7	3582	hydroxyl-moiety	1585	C-C stretch
			1661	C=O
			1828	C=C
			2535	CO_2_ stretching vibrations
			3640	O-H

^1^ Functional moieties of Ga-doped CuO NPs at minimum plant concentration. ^2^ Functional groups of Ga-doped CuO NPs at maximum plant concentration.

**Table 3:** Bactericidal action of CuO NPs.

Microorganism	Sample	^1^ Inhibition Zone (mm)		^2^ Inhibition Zone (mm)	
		500 μg/50 μL	1000 μg/50 μL	500 μg/50 μL	1000 μg/50 μL
MDR *S. aureus*	(1.2 mL:1) 1	2.05	3.15	1.1	1.25
	(1.8 mL:1) 2	2.45	3.45	2.15	2.4
	(2.4 mL:1) 3	2.8	3.8	2.3	2.7
	(3.0 mL:1) 4	3.15	4.5	2.8	3.05
	(3.6 mL:1) 5	3.8	5.65	3.55	4.45
	(4.2 mL:1) 6	3.35	5.35	3.4	4.05
	Ciprofloxacin	7.5	7.5	7.5	7.5
	DIW	0	0	0	0

^1^ Inhibition zones of CuO NPs fabricated with Gi extract. ^2^ Inhibition areas (mm) of NPs fabricated with Ga extract.

**Table 4:** Surflex score of docked ligand CuO.

Proteins	Docking Complex	CScore ^a^	Crash Score ^b^	Polar Score ^c^	G Score ^d^	PMF Score ^e^	D score ^f^	Chem Score ^g^	Amino Acid Interaction
DHFR	CuO	5.18	−0.04	4.63	−42.4	0.257	−285.8	−3.7	I14, T12, Q95, F92, Y98, T46, T121
TyrRS	CuO	6.77	−0.11	6.41	−37.05	6.409	−338.9	−3.7	Q190, Y36, D177, Q174
ddlB	CuO	6.08	−0.23	5.59	−90.86	14.03	−217.0	−2.09	N308, D293, E222, E220, N305
DHPS	CuO	4.08	−0.06	4.78	−32.55	2.892	−160.6	−0.36	A173, G131, N130, N132

^a^ CScore is a consensus scoring system that ranks a ligand’s affinity using a variety of scoring algorithms. ^b^ Crash-score indicating inadvertent entry into the binding site. ^c^ The ligand’s polar area. ^d^ G-score indicating the energies of hydrogen bonding, complex (ligand-protein), and internal (ligand-ligand) interactions. ^e^ PMF-score denotes the Helmholtz free energies of interactions between protein-ligand atom pairs (Potential of Mean Force, PMF). ^f^ D-score denotes charge and van der Waals interactions between the protein and ligand. ^g^ Hydrogen bonding, lipophilic contact, and rotational entropy are all assigned chem-score points, along with an intercept term.

## Data Availability

Data available on request.

## Supplementary Materials

The following supporting information can be downloaded at: https://www.mdpi.com/article/10.3390/ijms23042335/s1.

## References

[1] Dodd F. (1983). Mastitis—progress on control. J. Dairy Sci..

[2] Rinaldi M., Li R.W., Capuco A.V. (2010). Mastitis associated transcriptomic disruptions in cattle. Vet. Immunol. Immunopathol..

[3] Hussain R., Javed M.T., Khan A., Mahmood F., Kausar R. (2012). Mastitis and associated histo-pathological consequences in the context of udder morphology. Int. J. Agric. Biol..

[4] Hussain R., Khan A., Javed M.T., Ali F. (2013). Morphometric and pathological studies on mammary gland of slaughtered Nili-Ravi buffaloes. Pak. J. Agri. Sci..

[5] Oliver S., Gonzalez R., Hogan J., Jayarao B., Owens W. (2004). Microbiological Procedures for the Diagnosis of Bovine Udder Infection and Determination of Milk Quality.

[6] Hawkey P. (2008). The growing burden of antimicrobial resistance. J. Antimicrob. Chemother..

[7] Komolafe O. (2003). Antibiotic resistance in bacteria-an emerging public health problem. Malawi Med. J..

[8] Lowy F.D. (1998). *Staphylococcus aureus* infections. N. Engl. J. Med..

[9] Bradley A., Leach K., Breen J., Green L., Green M. (2007). Survey of the incidence and aetiology of mastitis on dairy farms in England and Wales. Vet. Record..

[10] Barkema H., Schukken Y., Zadoks R. (2006). Invited review: The role of cow, pathogen, and treatment regimen in the therapeutic success of bovine *Staphylococcus aureus* mastitis. J. Dairy Sci..

[11] Paterson G.K., Harrison E.M., Holmes M.A. (2014). The emergence of mecC methicillin-resistant *Staphylococcus aureus*. Trends Microbiol..

[12] Barkema H.W., Green M., Bradley A.J., Zadoks R. (2009). Invited review: The role of contagious disease in udder health. J. Dairy Sci..

[13] Brady R.A., Graeme A., Leid J.G., Prior M.L., Costerton J.W., Shirtliff M.E. (2011). Resolution of *Staphylococcus aureus* biofilm infection using vaccination and antibiotic treatment. Infect. Immun..

[14] Kenar B., Kuyucuoğlu Y., Şeker E. (2012). Antibiotic susceptibility of coagulase-negative *staphylococci* isolated from bovine subclinical mastitis in Turkey. Pak. Vet. J..

[15] Declercq P., Petre D., Gordts B., Voss A. (2008). Complicated community-acquired soft tissue infection by MRSA from porcine origin. Infection.

[16] Witte W., Strommenger B., Stanek C., Cuny C. (2007). Methicillin-resistant *Staphylococcus aureus* ST398 in humans and animals, Central Europe. Emerg. Infect. Dis..

[17] Nunan C., Young R. (2007). MRSA in Farm Animals and Meat, a New Threat to Human Health.

[18] Elhaig M.M., Selim A., Mahmoud M.M., El-Gayar E.K. (2016). Molecular confirmation of Trypanosoma evansi and Babesia bigemina in cattle from lower Egypt. Pak. Vet. J..

[19] Qayyum A., Khan J.A., Hussain R., Avais M., Ahmad N., Khan M.S. (2016). Investigation of milk and blood serum biochemical profile as an indicator of sub-clinical mastitis in Cholistani cattle. Pak. Vet. J..

[20] Yilmaz R., Cangul I., Onat K., Akkoc A., Ozyigit M., Akdesir E. (2016). Histopathological, immunohistochemical and bacteriological characterization of Mycoplasma bovis pneumonia in cattle. Pak. Vet. J..

[21] Adesiyun A.A., Webb L., Romain H.T. (1998). Prevalence and characteristics of *Staphylococcus aureus* strains isolated from bulk and composite milk and cattle handlers. J. Food Prot..

[22] Jahan M., Rahman M., Parvej M.S., Chowdhury S.M.Z.H., Haque M.E., Talukder M.A.K., Ahmed S. (2015). Isolation and characterization of *Staphylococcus aureus* from raw cow milk in Bangladesh. J. Adv. Vet. Anim. Res..

[23] Zecconi A., Piccinini R. (1998). *Staphilococcus aureus*: A problem for Italian dairy herds. Int. Dairy Fed..

[24] Alonzo F., Benson M.A., Chen J., Novick R.P., Shopsin B., Torres V.J. (2012). *Staphylococcus aureus* leucocidin ED contributes to systemic infection by targeting neutrophils and promoting bacterial growth in vivo. Mol. Microbiol..

[25] Kreger A.S., Kim K.S., Zaboretzky F., Bernheimer A.W. (1971). Purification and properties of staphylococcal delta hemolysin. Infect. Immun..

[26] Malachowa N., Kobayashi S.D., Braughton K.R., Whitney A.R., Parnell M.J., Gardner D.J., DeLeo F.R. (2012). *Staphylococcus aureus* leukotoxin GH promotes inflammation. J. Infect. Dis..

[27] Ventura C.L., Malachowa N., Hammer C.H., Nardone G.A., Robinson M.A., Kobayashi S.D., DeLeo F.R. (2010). Identification of a novel *Staphylococcus aureus* two-component leukotoxin using cell surface proteomics. PLoS ONE.

[28] Wang R., Braughton K.R., Kretschmer D., Bach T.H.L., Queck S.Y., Li M., Kennedy A.D., Dorward D.W., Klebanoff S.J., Peschel A. (2007). Identification of novel cytolytic peptides as key virulence determinants for community-associated MRSA. Nat. Med..

[29] Kobayashi S.D., Malachowa N., DeLeo F.R. (2015). Pathogenesis of *Staphylococcus aureus* abscesses. Am. J. Pathol..

[30] Raj A., Lawrence R.S., Jalees M., Lawrence K. (2015). Anti-bacterial activity of zinc oxide nanoparticles prepared from *Brassica oleraceae* leaves extract. Int. J. Adv. Res..

[31] Van den Bogaard A.E., Stobberingh E.E. (2000). Epidemiology of resistance to antibiotics: Links between animals and humans. Int. J. Antimicrob. Agents.

[32] Allahverdiyev A.M., Abamor E.S., Bagirova M., Rafailovich M. (2011). Antimicrobial effects of TiO_2_ and Ag_2_O nanoparticles against drug-resistant bacteria and leishmania parasites. Future Microbiol..

[33] Padnya P., Gorbachuk V., Stoikov I. (2020). The Role of Calix [n] arenes and Pillar [n] arenes in the Design of Silver Nanoparticles: Self-Assembly and Application. Int. J. Mol. Sci..

[34] Khatami M., Alijani H.Q., Heli H., Sharifi I. (2018). Rectangular shaped zinc oxide nanoparticles: Green synthesis by Stevia and its biomedical efficiency. Ceram. Int..

[35] Drummer S., Madzimbamuto T., Chowdhury M. (2021). Green Synthesis of Transition-Metal Nanoparticles and Their Oxides: A Review. Materials.

[36] Asghari F., Jahanshiri Z., Imani M., Shams-Ghahfarokhi M., Razzaghi-Abyaneh M. (2016). Antifungal nanomaterials: Synthesis, properties, and applications. Nanobiomaterials in Antimicrobial Therapy.

[37] Liu H., Zheng S., Xiong H., Alwahibi M.S., Niu X. (2020). Biosynthesis of copperoxide nanoparticles using Abies spectabilis plant extract and analyzing its antinociceptive and anti-inflammatory potency in various mice models. Arab. J. Chem..

[38] Ijaz F., Shahid S., Khan S.A., Ahmad W., Zaman S. (2017). Green synthesis of copper oxide nanoparticles using Abutilon indicum leaf extract: Antimicrobial, antioxidant and photocatalytic dye degradation activitie. Trop. J. Pharm. Res..

[39] Dey A., Manna S., Chattopadhyay S., Mondal D., Chattopadhyay D., Raj A., Das S., Bag B.G., Roy S. (2019). *Azadirachta indica* leaves mediated green synthesized copper oxide nanoparticles induce apoptosis through activation of TNF-α and caspases signaling pathway against cancer cells. J. Saudi Chem. Soc..

[40] Aminuzzaman M., Kei L., Liang W. (2017). Green and Sustainable Technology. AIP Conf. Proc..

[41] Shanan Z.J., Hadi S.M., Shanshool S.K. (2018). Structural analysis of chemical and green synthesis of CuO nanoparticles and their effect on biofilm formation. Baghdad Sci. J..

[42] Keabadile O.P., Aremu A.O., Elugoke S.E., Fayemi O.E. (2020). Green and Traditional Synthesis of Copper Oxide Nanoparticles—Comparative Study. Nanomaterials.

[43] Chouke P., Potbhare A., Dadure K., Mungole A., Meshram N., Chaudhary R., Rai A., Chaudhary R. (2020). An antibacterial activity of Bauhinia racemosa assisted ZnO nanoparticles during lunar eclipse and docking assay. Mater. Today Proc..

[44] Chouke P.B., Potbhare A.K., Bhusari G.S., Somkuwar S., Shaik D.P., Mishra R.K., Chaudhary R.G. (2019). Green fabrication of Zinc oxide nanospheres by Aspidopterys Cordata for effective antioxidant and antibacterial activity. Adv. Mater Lett..

[45] Król A., Pomastowski P., Rafińska K., Railean-Plugaru V., Buszewski B. (2017). Zinc oxide nanoparticles: Synthesis, antiseptic activity and toxicity mechanism. Adv. Colloid Interface Sci..

[46] Jamdagni P., Khatri P., Rana J. (2018). Green synthesis of zinc oxide nanoparticles using flower extract of Nyctanthes arbor-tristis and their antifungal activity. J. King Saud. Univ. Sci..

[47] Vinayagam R., Selvaraj R., Arivalagan P., Varadavenkatesan T. (2020). Synthesis, characterization and photocatalytic dye degradation capability of Calliandra haematocephala-mediated zinc oxide nanoflowers. J. Photochem. Photobiol. B Biol..

[48] Mary A.A., Ansari A.T., Subramanian R. (2019). Sugarcane juice mediated synthesis of copper oxide nanoparticles, characterization and their antibacterial activity. J. King Saud Univ. Sci..

[49] Kargar M., Ghashang M., Mohammad Shafiee M. (2015). An Efficient Green Synthesis of Copper Oxide NanoCrystalline. Int. J. Bio-Inorg. Hybrid Nanomater..

[50] Cuong H.N., Pansambal S., Ghotekar S., Oza R., Hai N.T.T., Viet N.M., Nguyen V.H. (2022). New frontiers in the plant extract mediated biosynthesis of copper oxide (CuO) nanoparticles and their potential applications: A review. Environ. Res..

[51] Maham M., Sajadi S.M., Kharimkhani M.M., Nasrollahzadeh M. (2017). Biosynthesis of the CuO nanoparticles using Euphorbia Chamaesyce leaf extract and investigation of their catalytic activity for the reduction of 4-nitrophenol. IET Nanobiotechnol..

[52] Bordbar M., Sharifi-Zarchi Z., Khodadadi B. (2017). Green synthesis of copper oxide nanoparticles/clinoptilolite using Rheum palmatum L. root extract: High catalytic activity for reduction of 4-nitro phenol, rhodamine B., and methylene blue. J. Sol-Gel Sci. Technol..

[53] Nasrollahzadeh M., Maham M., Sajadi S.M. (2015). Green synthesis of CuO nanoparticles by aqueous extract of Gundelia tournefortii and evaluation of their catalytic activity for the synthesis of N-monosubstituted ureas and reduction of 4-nitrophenol. J. Colloid Interface Sci..

[54] Patel V.K., Bhattacharya S. (2017). Solid state green synthesis and catalytic activity of CuO nanorods in thermal decomposition of potassium periodate. Mater. Res. Express.

[55] Nasrollahzadeh M., Sajadi S.M., Rostami-Vartooni A., Hussin S.M. (2016). Green synthesis of CuO nanoparticles using aqueous extract of Thymus vulgaris L. leaves and their catalytic performance for N-arylation of indoles and amines. J. Colloid Interface Sci..

[56] Patil A.S., Patil M.D., Lohar G.M., Jadhav S.T., Fulari V.J. (2017). Supercapacitive properties of CuO thin films using modified SILAR method. Ionics.

[57] Pansambal S., Deshmukh K., Savale A., Ghotekar S., Pardeshi O., Jain G., Aher Y., Pore D. (2017). Phytosynthesis and biological activities of fluorescent CuO nanoparticles using *Acanthospermum hispidum* L. extract. J. Nanostructures.

[58] Baig N., Saleh T.A. (2019). Superhydrophobic Polypropylene Functionalized with Nanoparticles for Efficient Fast Static and Dynamic Separation of Spilled Oil from Water. Glob. Chall..

[59] Ghosh S., Kundu S., Naskar M.K. (2021). Mesoporous CuO nanostructures for low-temperature CO oxidation. Bull. Mater. Sci..

[60] Hamza M.F., Wei Y., Mira H., Adel A.H., Guibal E. (2019). Synthesis and adsorption characteristics of grafted hydrazinyl amine magnetite-chitosan for Ni (II) and Pb (II) recovery. Chem. Eng. J..

[61] Jurado-López B., Vieira R.S., Rabelo R.B., Beppu M.M., Casado J., Rodríguez-Castellón E. (2017). Formation of complexes between functionalized chitosan membranes and copper: A study by angle resolved XPS. Mater. Chem. Phys..

[62] Ortega-Liebana M., Chung N., Limpens R., Gomez L., Hueso J., Santamaria J., Gregorkiewicz T. (2017). Uniform luminescent carbon nanodots prepared by rapid pyrolysis of organic precursors confined within nanoporous templating structures. Carbon.

[63] Poulston S., Parlett P., Stone P., Bowker M. (1996). Surface oxidation and reduction of CuO and Cu2O studied using XPS and XAES. Surf. Interface Anal..

[64] Brakstad O.G., Aasbakk K., Maeland J.A. (1992). Detection of *Staphylococcus aureus* by polymerase chain reaction amplification of the nuc gene. J. Clin. Microbiol..

[65] Jesudoss S.K., Vijaya J.J., Kennedy L.J., Rajan P.I., Al-Lohedan A.H., Ramalingam R.J., Kaviyarasu K., Bououdina M. (2016). Studies on the efficient dual performance of Mn1–xNixFe2O4 spinel nanoparticles in photodegradation and antibacterial activity. J. Photochem. Photobio. B Biol..

[66] Navale G.R., Rout C.S., Gohil K.N., Dharne M.S., Late D.J., Shinde S.S. (2015). Oxidative and membrane stress-mediated antibacterial activity of ws 2 and rgo-ws 2 nanosheets. Rsc Adv..

[67] Ahmed B., Hashmi A., Khan M.S., Musarrat J. (2018). ROS mediated destruction of cell membrane, growth and biofilms of human bacterial pathogens by stable metallic AgNPs functionalized from bell pepper extract and quercetin. Adv. Powder Technol..

[68] Ahmed B., Solanki B., Zaidi A., Khan M.S., Musarrat J. (2019). Bacterial toxicity of biomimetic green zinc oxide nanoantibiotic: Insights into ZnONP uptake and nanocolloid–bacteria interface. Toxicol. Res..

[69] Ali K., Ahmed B., Ansari S.M., Saquib Q., Al-Khedhairy A.A., Dwivedi S., Alshaeri M., Khan M.S., Musarrat J. (2019). Comparative in situ ROS mediated killing of bacteria with bulk analogue, Eucalyptus leaf extract (ELE)-capped and bare surface copper oxide nanoparticles. Mater. Sci. Eng. C.

[70] Ali K., Ahmed B., Khan M.S., Musarrat J. (2018). Differential surface contact killing of pristine and low EPS Pseudomonas aeruginosa with Aloe vera capped hematite (α-Fe2O3) nanoparticles. J. Photochem. Photobiol. B Biol..

[71] Haider A., Ijaz M., Imran M., Naz M., Majeed H., Khan J., Ali M., Ikram M. (2020). Enhanced bactericidal action and dye degradation of spicy roots’ extract-incorporated fine-tuned metal oxide nanoparticles. Appl. Nanosci..

[72] Haroon M., Zaidi A., Ahmed B., Rizvi A., Khan M.S., Musarrat J. (2019). Effective inhibition of phytopathogenic microbes by eco-friendly leaf extract mediated silver nanoparticles (AgNPs). Indian J. Microbiol..

[73] Haider A., Ijaz M., Ali S., Haider J., Imran M., Majeed H., Shahzadi I., Ali M.M., Khan J.A., Ikram M. (2020). Green synthesized phytochemically (Zingiber officinale and Allium sativum) reduced nickel oxide nanoparticles confirmed bactericidal and catalytic potential. Nanoscale Res. Lett..

[74] Raja P.I., Vijaya J.J., Jesudoss S., Kaviyarasu K., Kennedy L.J., Jothiramalingam R., Al-Lohedan H.A., Vaali-Mohammed M-A (2017). Green-fuel-mediated synthesis of self-assembled NiO nano-sticks for dual applications—Photocatalytic activity on Rose Bengal dye and antimicrobial action on bacterial strains. Mater. Res. Express.

[75] Jayawardena H.S.N., Jayawardana K.W., Chen X., Yan M. (2013). Maltoheptaose promotes nanoparticle internalization by Escherichia coli. Chem. Commun..

[76] Fatimah I., Aftrid Z.H.V.I. (2019). Characteristics and antibacterial activity of green synthesized silver nanoparticles using red spinach (*Amaranthus Tricolor*, L.) leaf extract. Green Chem. Lett. Rev..

[77] David S.A., Rajadurai S.I., Kumar S.V. (2017). Biosynthesis of copper oxide nanoparticles using Momordica charantia leaf extract and their characterization. Int. J. Adv. Res. Sci. Eng..

[78] Ren G., Hu D., Cheng E.W., Vargas-Reus M.A., Reip P., Allaker R.P. (2009). Characterisation of copper oxide nanoparticles for antimicrobial applications. Int. J. Antimicrob. Agents.

[79] Nabila M.I., Kannabiran K. (2018). Biosynthesis, characterization and antibacterial activity of copper oxide nanoparticles (CuO NPs) from actinomycetes. Biocatal. Agric. Biotechnol..

[80] Bigdeli F., Morsali A., Retailleau P. (2010). Syntheses and characterization of different zinc (II) oxide nano-structures from direct thermal decomposition of 1D coordination polymers. Polyhedron.

[81] Ganesan S., Babu I.G., Mahendran D., Arulselvi P.I., Elangovan N., Geetha N., Venkatachalam P. (2016). Green engineering of titanium dioxide nanoparticles using *Ageratina altissima* (L.) King & HE Robines. medicinal plant aqueous leaf extracts for enhanced photocatalytic activity. Ann. Phytomed.

[82] Jafarirad S., Mehrabim M., Divband B., Kosari-Nasab M. (2016). Biofabrication of zinc oxide nanoparticles using fruit extract of Ros*a canina* and their toxic potential against bacteria: A mechanistic approach. Mater. Sci. Eng. C.

[83] Jayaprakash J., Srinivasan N., Chandrasekaran P., Girija E. (2015). Synthesis and characterization of cluster of grapes like pure and Zinc-doped CuO nanoparticles by sol–gel method. Spectrochim. Acta Part A Mol. Biomol. Spectrosc..

[84] Velsankar K., Kumar A.R.M., Preethi R., Muthulakshmi V., Sudhahar S. (2020). Green synthesis of CuO nanoparticles via Allium sativum extract and its characterizations on antimicrobial, antioxidant, antilarvicidal activities. J. Environ. Chem. Eng..

[85] Shankar S.S., Rai A., Ahmad A., Sastry M. (2004). Rapid synthesis of Au, Ag, and bimetallic Au core–Ag shell nanoparticles using Neem (*Azadirachta indica*) leaf broth. J. Colloid Interface Sci..

[86] Rasul Suleria H.A., Sadiq Butt M., Muhammad Anjum F., Saeed F., Batool R., Nisar Ahmad A. (2012). Aqueous garlic extract and its phytochemical profile; special reference to antioxidant status. Int. J. Food Sci. Nutr..

[87] Salam H.A., Sivaraj R., Venckatesh R. (2014). Green synthesis and characterization of zinc oxide nanoparticles from Ocimum basilicum L. var. purpurascens Benth-Lamiaceae leaf extract. Mater. Lett..

[88] Selvarajan E., Mohanasrinivasan V. (2013). Biosynthesis and characterization of ZnO nanoparticles using *Lactobacillus plantarum* VITES07. Mater. Lett..

[89] Yang Y., Matsubara S., Nogami M., Shi J. (2007). Controlling the aggregation behavior of gold nanoparticles. Mater. Sci. Eng. B.

[90] Yang J.A., Lohse S.E., Murphy C.J. (2014). Tuning cellular response to nanoparticles via surface chemistry and aggregation. Small.

[91] Hood M.A., Mari M., Muñoz-Espí R. (2014). Synthetic strategies in the preparation of polymer/inorganic hybrid nanoparticles. Materials.

[92] Larmagnac A., Eggenberger S., Janossy H., Vörös J. (2014). Stretchable electronics based on Ag-PDMS composites. Sci. Rep..

[93] Mallakpour S., Zeraatpisheh F. (2012). Preparation and morphology distinguishing of novel ZnO ultrafine particle filled nanocomposites contain new poly (*amide-imide*) via ultrasonic process. J. Polym. Res..

[94] Gudding R.O.A.R. (1983). Differentiation of *staphylococci* on the basis of nuclease properties. J. Clin. Microbiol..

[95] Victor R., Lachica F., Jang S.S., Hoeprich P.D. (1979). Thermonuclease seroinhibition test for distinguishing *Staphylococcus aureus* from other coagulase-positive *staphylococci*. J. Clin. Microbiol..

[96] Brakstad O.G., Maeland J.A. (1989). Generation and characterization of monoclonal antibodies against *Staphylococcus.aureus*. Acta Pathol. Microbiol. Immunol. Scand..

[97] Liebl W., Rosenstein R., Götz F., Schleifer K.H. (1987). Use of staphylococcal nuclease gene as DNA probe for *Staphylococcus aureus*. FEMS Microbiol. Lett..

[98] Shortle D. (1983). A genetic system for analysis of staphylococcal nuclease. Gene.

[99] Xiao Z.P., Ma T.W., Liao M.L., Feng Y.T., Peng X.C., Li J.L., Li Z.P., Wu Y., Luo Q., Deng Y. (2011). Tyrosyl-tRNA synthetase inhibitors as antibacterial agents: Synthesis, molecular docking and structure–activity relationship analysis of 3-aryl-4-arylaminofuran-2 (5H)-ones. Eur. J. Med. Chem..

[100] Hawser S., Lociuro S., Islam K. (2006). Dihydrofolate reductase inhibitors as antibacterial agents. Biochem. Pharmacol..

[101] Hitchings G.H., Burchall J.J. (1965). Inhibition of folate biosynthesis and function as a basis for chemotherapy. Adv. Enzymol. Relat. Areas Mol. Biol..

[102] Indana M.K., Gangapuram B.R., Dadigala R., Bandi R., Guttena V. (2016). A novel green synthesis and characterization of silver nanoparticles using gum tragacanth and evaluation of their potential catalytic reduction activities with methylene blue and Congo red dyes. J. Anal. Sci. Technol..

[103] Ganapuram B.R., Alle M., Dadigala R., Dasari A., Maragoni V., Guttena V. (2015). Catalytic reduction of methylene blue and Congo red dyes using green synthesized gold nanoparticles capped by salmalia malabarica gum. Int. Nano Lett..

[104] Saha J., Begum A., Mukherjee A., Kumar S. (2017). A novel green synthesis of silver nanoparticles and their catalytic action in reduction of Methylene Blue dye. Sustain. Environ. Res..

[105] Sutradhar P., Saha M., Maiti D. (2014). Microwave synthesis of copper oxide nanoparticles using tea leaf and coffee powder extracts and its antibacterial activity. J. Nanostructure Chem..

[106] Veisi H., Karmakar B., Tamoradi T., Hemmati S., Hekmati M., Hamelian M. (2021). Biosynthesis of CuO nanoparticles using aqueous extract of herbal tea (*Stachys Lavandulifolia*) flowers and evaluation of its catalytic activity. Sci. Rep..

[107] Iwalokun B., Ogunledun A., Ogbolu D., Bamiro S., Jimi-Omojola J. (2004). In vitro antimicrobial properties of aqueous garlic extract against multidrug-resistant bacteria and Candida species from Nigeria. J. Med. Food.

[108] Cherian T., Ali K., Saquib Q., Faisal M., Wahab R., Musarrat J. (2020). Cymbopogon citratus functionalized green synthesis of CuO-nanoparticles: Novel prospects as antibacterial and antibiofilm agents. Biomolecules.

[109] Pisano M.B., Kumar A., Medda R., Gatto G., Pal R., Fais A., Era B., Cosentino S., Uriarte E., Santana L. (2019). Antibacterial activity and molecular docking studies of a selected series of hydroxy-3-arylcoumarins. Molecules.

[110] Oefner C., Parisi S., Schulz H., Lociuro S., Dale G.E. (2009). Inhibitory properties and X-ray crystallographic study of the binding of AR-101, AR-102 and iclaprim in ternary complexes with NADPH and dihydrofolate reductase from *Staphylococcus aureus*. Acta Crystallogr. Sect. D Biol. Crystallogr..

[111] Liu S., Chang J.S., Herberg J.T., Horng M.M., Tomich P.K., Lin A.H., Marotti K.R. (2006). Allosteric inhibition of *Staphylococcus aureusd-alanine*: D-alanine ligase revealed by crystallographic studies. Proc. Natl. Acad. Sci. USA.

[112] Rehman K., Chohan T.A., Waheed I., Gilani Z., Akash M.S.H. (2019). Taxifolin prevents postprandial hyperglycemia by regulating the activity of α-amylase: Evidence from an in vivo and in silico studies. J. Cell. Biochem..

[113] Jain A.N. (2003). Surflex: Fully automatic flexible molecular docking using a molecular similarity-based search engine. J. Med. Chem..

[114] Clark M., Cramer R.D., Van Opdenbosch N. (1989). Validation of the general purpose Tripos 5.2 force field. J. Comput. Chem..

[115] Jain A.N. (1996). Scoring noncovalent protein-ligand interactions: A continuous differentiable function tuned to compute binding affinities. J. Comput. Aided Mol. Des..

[116] Welch W., Ruppert J., Jain A.N. (1996). Hammerhead: Fast, fully automated docking of flexible ligands to protein binding sites. Chem. Biol..

