# Supplementary figures and images for: In-Vitro Catalytic and Antibacterial Potential of Green Synthesized CuO Nanoparticles against Prevalent Multiple Drug Resistant Bovine Mastitogen Staphylococcus aureus
Source: Int J Mol Sci. 2022 Feb 20;23(4):2335. doi: 10.3390/ijms23042335 (PMC8878101; doi:10.3390/ijms23042335)

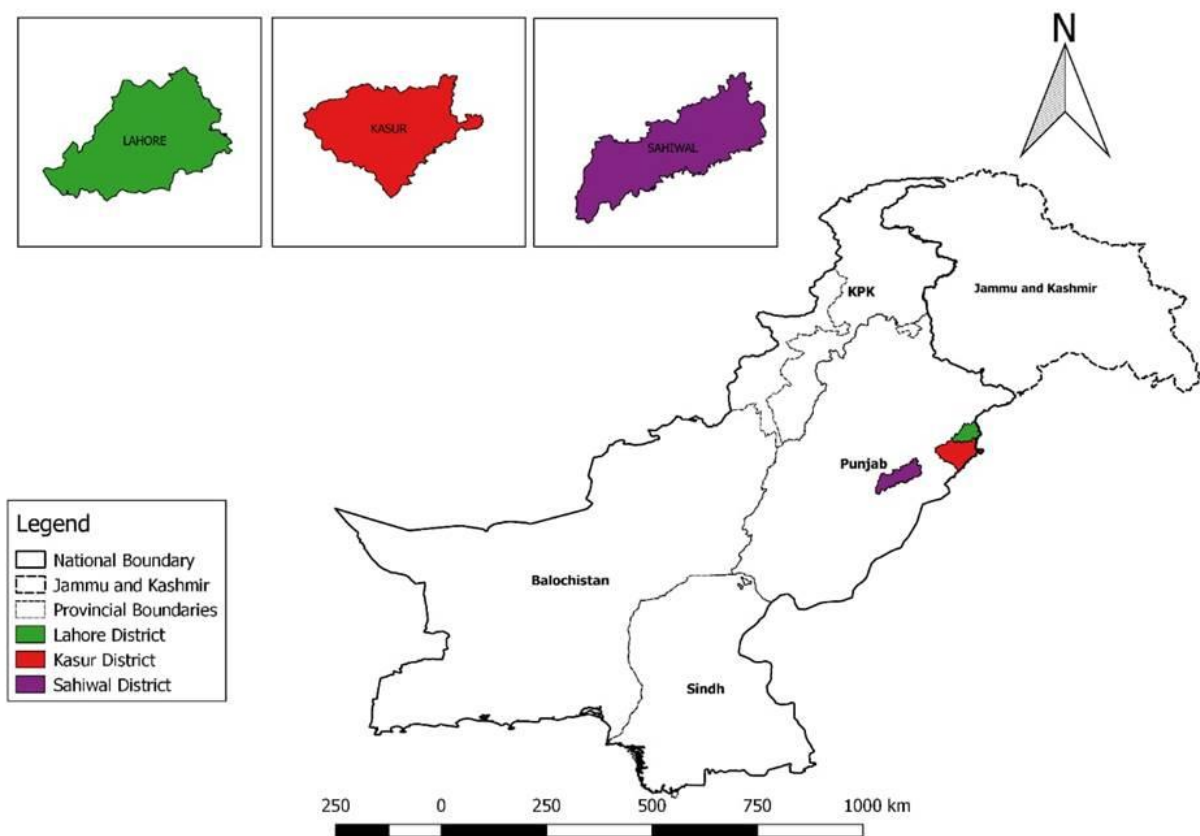

**Figure S1.** Sample collection sites indicated by different colors.

Supplement: Supplementary file 1 [file ijms-23-02335-s001.zip › ijms-1572266-supplementary.pdf]
